# A bibliometric analysis of childhood immunization research productivity in Africa since the onset of the Expanded Program on Immunization in 1974

**DOI:** 10.1186/1741-7015-11-66

**Published:** 2013-03-14

**Authors:** Charles S Wiysonge, Olalekan A Uthman, Peter M Ndumbe, Gregory D Hussey

**Affiliations:** 1 Vaccines for Africa Initiative, Institute of Infectious Disease and Molecular Medicine, University of Cape Town, Anzio Road Observatory, Cape Town 7925, South Africa; 2 Division of Medical Microbiology, Department of Clinical Laboratory Sciences, University of Cape Town, Anzio Road Observatory, Cape Town 7925, South Africa; 3 Centre for Applied Health Research and Delivery, University of Warwick, Warwick Medical School, Gibbet Hill Rd, Coventry, CV4 7AL, UK; 4 International Health Group, Liverpool School of Tropical Medicine, Pembroke Place, Liverpool, L3 5QA, UK; 5 Research, Publications and Library Services/Health Systems and Services Cluster, WHO Regional Office for Africa, PO Box 6, Djoue-Brazzaville, Congo

**Keywords:** Africa, bibliometrics, childhood immunization, Expanded Program on Immunization, low and middle-income counries, research, sub-Saharan Africa

## Abstract

**Background:**

The implementation of strategic immunization plans whose development is informed by available locally-relevant research evidence should improve immunization coverage and prevent disease, disability and death in Africa. In general, health research helps to answer questions, generate the evidence required to guide policy and identify new tools. However, factors that influence the publication of immunization research in Africa are not known. We, therefore, undertook this study to fill this research gap by providing insights into factors associated with childhood immunization research productivity on the continent. We postulated that research productivity influences immunization coverage.

**Methods:**

We conducted a bibliometric analysis of childhood immunization research output from Africa, using research articles indexed in PubMed as a surrogate for total research productivity. We used zero-truncated negative binomial regression models to explore the factors associated with research productivity.

**Results:**

We identified 1,641 articles on childhood immunization indexed in PubMed between 1974 and 2010 with authors from Africa, which represent only 8.9% of the global output. Five countries (South Africa, Nigeria, The Gambia, Egypt and Kenya) contributed 48% of the articles. After controlling for population and gross domestic product, The Gambia, Guinea-Bissau and Sao Tome and Principe were the most productive countries. In univariable analyses, the country's gross domestic product, total health expenditure, private health expenditure, and research and development expenditure had a significant positive association with increased research productivity. Immunization coverage, adult literacy rate, human development index and physician density had no significant association. In the multivarable model, only private health expenditure maintained significant statistical association with the number of immunization articles.

**Conclusions:**

Immunization research productivity in Africa is highly skewed, with private health expenditure having a significant positive association. However, the current contribution of authors from Africa to global childhood immunization research output is minimal. The lack of association between research productivity and immunization coverage may be an indication of lack of interactive communication between health decision-makers, program managers and researchers; to ensure that immunization policies and plans are always informed by the best available evidence.

## Background

The routine childhood immunization program was launched by the World Health Organization (WHO) in 1974, following the successful program for the eradication of small pox [1]. The program, known as the Expanded Program on Immunization (or EPI), consists of regularly scheduled services that reach each new cohort of children less than one year of age with vaccines at health facilities, scheduled outreach sites, or (in special circumstances) from door to door. The program is composed of a series of inter-related components, including service delivery, vaccine supply and quality, logistics, advocacy and communication, surveillance, financing, management and capacity building. During the past four decades national EPI programs have developed or adapted and implemented a broad range of strategies and activities aimed at bringing services closer to the targeted community, increasing demand for immunization services, reaching previously unreached children, and improving immunization data quality [2-6]. Through these efforts, the mean proportion of the annual birth cohort that received a full series of three doses of the diphtheria, tetanus and pertussis vaccine (DTP3) reached 77% in sub-Saharan Africa in 2010 [7,8]. Ideally, the development or adaptation and implementation of these interventions should be informed by the best available local evidence [9-11]. The increase in childhood immunization coverage in Africa over the last four decades would, therefore, be expected to have been accompanied by similar growth in childhood immunization research from the continent.

Research publications have an important role in the scientific process providing a key linkage between knowledge generation, uptake and use [12-14]. For a long time, publications and their citation (that is, bibliometrics) have been the method of choice for quantitative assessments of academic research at international, national, institutional and individual levels [15-18]. Bibliometric analysis is also a feasible tool to comprehensively recognize the research advances in the past and future research trends in a specific field. In the context of the African continent to date, factors related to variation in immunization research productivity have not been examined, although bibliometric studies with data on Africa exist in other disciplines [16-30]. Therefore, this bibliometric study aims to fill some of the gaps in existing research by providing insights into the history and growth of childhood immunization research in Africa. We examined whether national immunization coverage or other country-level factors are associated with childhood immunization research productivity in Africa.

## Methods

### Data sources

We used childhood immunization research articles indexed in the PubMed database as a surrogate for total childhood immunization research productivity. We searched the database in November 2011 in order to obtain the childhood immunization research volume of each African country from 1 January 1974 to 31 December 2010. Articles originating from each country, published between 1974 and 2010 were generated by selecting the advanced-search option and then selecting the "publication date" field. Next, the "affiliation" field was searched for each country. The names of the countries were imputed in their different possible forms, for example, Cameroon and Cameroun for Cameroon. Some names of countries are also names of parts of other countries, for example, Niger is the name of a place in Nigeria. To avoid errors arising from this, appropriate commands were used (that is, (Niger (AD) NOT Nigeria)). We then combined this with childhood immunization search terms: ("Immunization"(Mesh) OR "Vaccination"(Mesh) OR "Immunization, Secondary"(Mesh) OR "Immunization Programs"(Mesh) OR "Immunization Schedule"(Mesh) OR "Immunization, Passive"(Mesh) OR "Mass Vaccination"(Mesh)) AND ("Infant, Newborn"(Mesh) OR "Infant"(Mesh) OR "Child, Preschool" (Mesh)).

The 2010 data on DTP3 coverage, adult literacy rate, gross domestic product (GDP), public expenditure on health (as a percentage of GDP), human development index, research and development expenditure, physicians (per 100,000 population), total expenditure on health and out-of-pocket health expenditure (that is, private expenditure on health) were obtained from the reports published by the WHO [7], United Nations Development Program and the World Bank [31].

### Statistical analyses

We calculated the ratios of the number of publications from countries to their population, GDP and health expenditure in order to allow weighted comparisons. We used Pearson's correlation analysis to examine the association between research productivity and GDP, health expenditure, and research and development expenditure. Factors associated with variation in childhood immunization research productivity were explored using univariable and multivariable regression models for count outcomes.

We used the Bayesian Information Criterion (BIC) to compare different count regression models [32-36]. The BIC assesses the overall fit of a model and allows the comparison of both nested and non-nested models. It is based on a Bayesian comparison of models [32-36]. In case of two or more count regression models, under the assumption of no prior preference for one model over the other, BIC identifies the model that is more likely to have generated the observed data [32-36].

The formula for the BIC statistic reported by Stata [36], (which *estat ic *labels as BIC used by Stata), is:

BIC = - 2*ln(likelihood) + ln(N)*k

where *N *is the number of observations used in estimation or the number of independent terms in the likelihood and *k *is the model degrees of freedom, number of parameter estimated including the constant calculated as the rank of variance-covariance matrix of the parameters. Given that the models fit on the same data, the model with the smallest value of the BIC is considered to be the best [32-36]. The most parsimonious model can be identified as the one with the lowest BIC, which in this case was the zero-truncated negative binomial regression (see Additional file 1). Univariable zero-truncated negative binomial regression analyses were used to investigate the bivariate relationship between each country-level factor listed above and total research productivity. Multivariable zero-truncated negative binomial regression analyses were carried out to determine which country-level factors were independently associated with total research productivity. Only variables with a value of *P *<0.2 in univariable analyses were included in the multivariable model. Results were presented as incidence rate ratios (IRR) with 95% confidence intervals (CIs) and percentage change. For zero-truncated negative binomial regression, country-level indicators were log transformed to linearize these associations. All tests were two-sided and statistical significance was defined at the 5% alpha level. Data were processed and analyzed with Stata 12 software (Stata Corp., College Station, TX, USA).

## Results

A total of 1,641 articles on childhood immunization indexed by PubMed between 1974 and 2010 are described in this study. The summary statistics for all country-level factors included in this study are shown in Table 1. The percentage of children that received DTP3 based on WHO/UNICEF 2010 estimates ranged from as low as 33% in Equatorial Guinea to 99% in Cape Verde, Eritrea, Mauritius, Morocco and Seychelles. The median adult literacy was 66% (range 26.2% to 93.0%). The median number of physicians per 100,000 population was 31 (range 2 to 243).

**Table 1:** Descriptive statistics of selected country-level variables

Variable	Median	Range
DTP3 coverage (%)	81.2	33.0 to 99.0
Gross domestic product (USD billions)	27.9	0.2 to 282.8
Adult literacy rate (%)	66.0	26.2 to 93.0
Physicians per 100,000 population	31.1	2.0 to 243.0
Total expenditure on health (% of GDP)	6.1	2.0 to 13.0
Private expenditure on health (% of GDP)	3.2	1.0 to 12.0
R&D expenditure (PPP, USD millions)	129.6	0.0 to 2494.0
Human development index	0.5	0.3 to 0.8

DTP3, third dose of the diphtheria-tetanus-pertussis vaccine by one year of age; USD, United States Dollar; R&D, research and development; PPP, purchasing power parity

**Figure 1:**
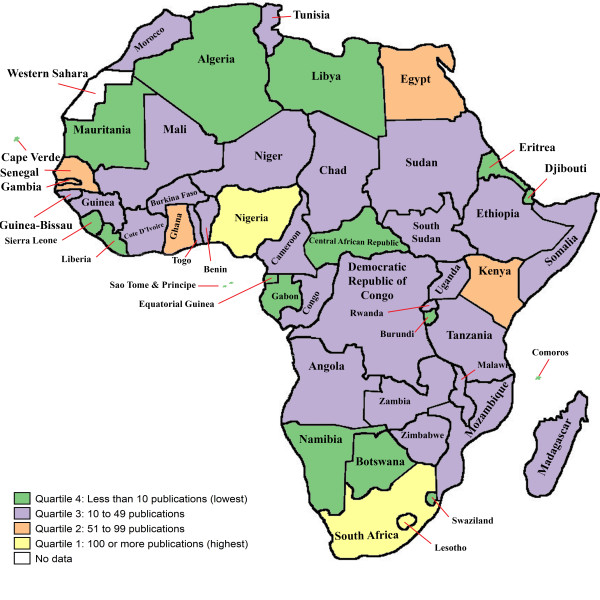
**Map of childhood immunization research articles from Africa indexed in PubMed, 1974 to 2010**.

The number of childhood immunization articles indexed in PubMed from each country is shown in Table 2. As shown in the table, Africa's publication output trends show that its contribution to global childhood immunization publications has been low during the period 1974 to 2010. The percentage share of global childhood immunization research output increased from 6.6% in 1974 to 1980 to 9.6% in 2001 to 2010. The median number of articles was 16 (range 1 to 346). Figure 1 shows the number of articles broken down by quartiles. Three countries (South Africa, Nigeria and The Gambia) are in the highest quartile with more than 100 articles. Four countries belong to the second quartile (50 to 99 articles) and 27 to the third quartile (10 to 49 articles). Twenty countries with less than 10 articles belong to the lowest quartile.

**Table 2:** Trends in childhood immunization articles output from Africa indexed by PubMed (1974 to 2010)

	Publications				
	
Country	1974 to 1980	1981 to 1990	1991 to 2000	2001 to 2010	1974 to 2010
**South Africa**	21	65	153	107	346
**Nigeria**	15	30	42	67	154
**Gambia**	1	20	43	40	104
**Egypt**	8	7	37	47	99
**Kenya**	11	16	24	36	87
**Senegal**	5	13	29	23	70
**Ghana**	11	4	13	26	54
**Zimbabwe**	1	14	22	9	46
**Ethiopia**	2	3	20	19	44
**Uganda**	6	7	10	17	40
**Burkina Faso**	0	5	11	24	40
**Tanzania**	1	8	9	21	39
**Malawi**	0	4	12	18	34
**Sudan**	1	11	11	10	33
**Zambia**	1	4	7	16	28
**Democratic Republic of Congo**	0	7	14	5	26
**Cameroon**	6	7	5	7	25
**Mali**	6	4	4	11	25
**Cote d`Ivoire**	2	6	8	9	25
**Mozambique**	0	8	3	13	24
**Morocco**	3	3	4	12	22
**Chad**	0	1	8	13	22
**Guinea-Bissau**	0	3	5	13	21
**Tunisia**	2	6	6	5	19
**Guinea**	0	0	10	9	19
**Togo**	2	3	6	7	18
**Angola**	0	0	10	6	16
**Niger**	1	0	6	8	15
**Madagascar**	1	1	6	7	15
**Somalia**	2	6	3	4	15
**Congo-Brazzaville**	0	4	5	6	15
**Benin**	0	1	4	8	13
**Rwanda**	0	1	6	5	12
**Namibia**	0	0	7	2	9
**Central African Republic**	1	0	5	1	7
**Algeria**	1	3	2	0	6
**Gabon**	0	1	3	2	6
**Liberia**	0	2	2	2	6
**Burundi**	0	0	4	1	5
**Sierra Leone**	1	1	3	0	5
**Djibouti**	0	1	2	1	4
**Botswana**	0	0	4	0	4
**Swaziland**	0	0	3	1	4
**Eritrea**	0	0	1	3	4
**Lesotho**	0	0	3	0	3
**Libya**	0	2	1	0	3
**Comoros**	0	0	1	1	2
**Cape Verde**	0	1	0	1	2
**Sao Tome and Principe**	0	1	1	0	2
**Seychelles**	0	0	1	0	1
**Mauritania**	0	1	0	0	1
**Mauritius**	0	0	1	0	1
**Equatorial Guinea**	0	0	1	0	1

**Total: Africa output**	112	285	601	643	1641
**Total: World output**	1,702	3,485	5,816	6,679	18,388
**% world research output**	6.6	8.2	10.3	9.6	8.9

**Figure 2:**
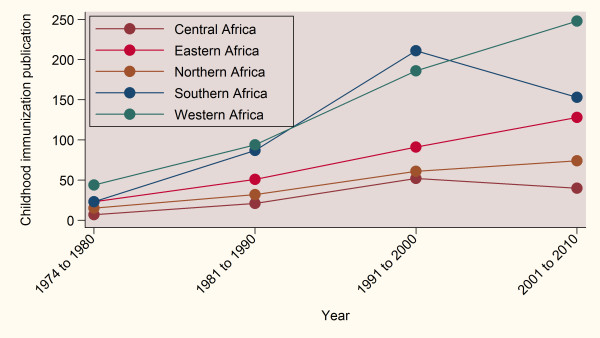
**Trends in Africa sub-regional childhood immunization research output indexed by PubMed, 1974 to 2010**.

Table 3 shows the top-ranking countries in terms of relative contribution of each country to the total number of articles. Authors from South Africa produced the highest number of articles (n = 346, 21%), followed by Nigeria (n = 154, 9%), and The Gambia (n = 104, 6%). In absolute terms, authors from the top five countries combined produced almost half (48%) of the total indexed articles. As shown in the table, The Gambia, Guinea-Bissau and Sao Tome and Principe had the highest number of publications after controlling for the country's population and GDP. When controlled for total expenditure on health, the top three countries were South Africa, Nigeria and Kenya. The trend in total production of childhood immunization articles in each geographical sub-region of Africa is displayed in Figure 2. West Africa was the most productive sub-region during the period studied. Apart from Central Africa and Southern Africa, which experienced a drop between 2001 and 2010, there was a continuous increase in the production of research articles from all African sub-regions during the period 1974 to 2010. The total number of articles from West Africa, for example, increased from 44 in 1974 to 1980 to 248 in 2001 to 2010.

**Table 3:** Top 10 African countries in terms of childhood immunization research productivity normalized by selected variables

Rank	Country	Number (%)	Population	Gross domestic product	Total expenditure on health
1	South Africa	346 (21.1)	The Gambia	The Gambia	South Africa
2	Nigeria	154 (9.4)	Guinea-Bissau	Guinea-Bissau	Nigeria
3	The Gambia	104 (6.3)	Sao Tome and Principe	Sao Tome and Principe	Kenya
4	Egypt	99 (6.0)	Seychelles	Zimbabwe	Egypt
5	Kenya	87 (5.3)	South Africa	Malawi	The Gambia
6	Senegal	70 (4.3)	Senegal	Liberia	Senegal
7	Ghana	54 (3.3)	Djibouti	Togo	Ethiopia
8	Zimbabwe	46 (2.8)	Cape Verde	Senegal	Tanzania
9	Ethiopia	44 (2.7)	Gabon	Burkina Faso	Ghana
10	Burkina Faso, and Uganda	40 (2.4)	Namibia	Guinea	Burkina Faso

**Figure 3:**
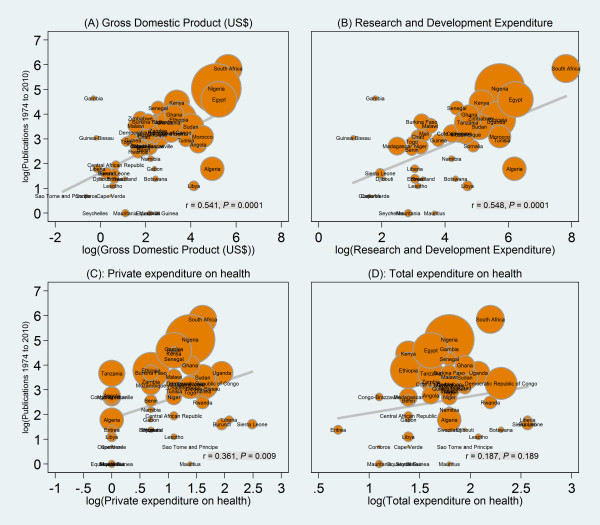
**Scatter plot showing association between the country's total immunization publications and selected country-level variables**.

Figure 3 shows the results of the correlation analyses. There was a strong positive and statistically significant correlation between the country's GDP (r = 0.541, *P *= 0.0001, Figure 3A), research and development expenditure (r = 0.548, *P *= 0.0001, Figure 3B), and total number of published articles on childhood immunization. Similarly, there was a moderately positive and statistically significant correlation between private expenditure on health and total number of articles (r = 0.361, *P *= 0.009, Figure 3C). However, there was no statistically significant correlation between total expenditure on health and immunization research productivity (r = 0.187, *P *= 0.189, Figure 3D).

Country-level factors associated with total childhood immunization research productivity are shown in Table 4. In the univariable model, a country's GDP, total expenditure on health, private expenditure on health, and research and development expenditure were statistically, significantly associated with increased childhood immunization research productivity. In the univariable analyses, immunization coverage, adult literacy rate, human development index and physician density had no significant statistical association with the number of immunization articles. As shown in the table, only private expenditure on health remained statistically significant in the multivariable model when all factors were controlled for statistically. Each unit increase in log dollar amount in private expenditure on health increased the total research productivity by 264% (IRR 3.64; 95% CI 1.46 to 9.07).

**Table 4:** Factors associated with childhood immunization research productivity identified by zero-truncated negative binomial regression models.

	Univariable		Multivariable	
**Variable**	**IRR (95% CI)**	***P*-value**	**IRR (95% CI)**	***P*-value**

DTP3 coverage	0.38 (0.04, 3.42)	0.389	not included	
Gross domestic product (US dollar)	1.46 (1.23, 1.73)	0.000	1.55 (0.82, 2.93)	0.177
Adult literacy rate	1.21 (0.36, 4.09)	0.764	not included	
Physicians per 100,000 population	1.27 (0.92, 1.74)	0.147	0.77 (0.56, 1.04)	0.089
Total expenditure on health	3.98 (0.97, 16.21)	0.054	0.68 (0.12, 3.94)	0.665
Private expenditure on health	3.18 (1.62, 6.22)	0.001	3.64 (1.46, 9.07)	0.006
Research and development expenditure	1.47 (1.21, 1.78)	0.000	0.96 (0.49, 1.87)	0.897
Human development index	2.65 (0.45, 15.57)	0.280	not included	

DTP3, third dose of the diphtheria-tetanus-pertussis vaccine by one year of age; IRR, incidence rate ratio; CI, confidence intervals

## Discussion

We found that childhood immunization research productivity in Africa is highly skewed. South Africa, Nigeria, The Gambia, Egypt and Kenya jointly account for almost half of the articles on childhood immunization indexed in PubMed between 1974 and 2010. There was a significant increase in the number of publications from all African sub-regions between 1974 and 2010. However, Africa's contribution to global childhood immunization publications has been minimal during the period studied. The Gambia, Guinea-Bissau, Sao Tome and Principe, Zimbabwe and Malawi had better records when the total research productivity was adjusted for gross domestic product. When controlled for total expenditure on health, South Africa, Nigeria, Kenya, Egypt and The Gambia were the most productive. Multiple medical schools and research institutions in South Africa, Nigeria and Egypt may account for the large number of publications from these three countries. Similarly, the presence of the British Medical Research Council, the Kenyan Medical Research Institute and a Danish research group (Bandim Health Project) may be the drivers of publications from The Gambia, Kenya and Guinea-Bissau, respectively.

In order to set our study in the context of other existing bibliometric studies, we searched PubMed, combining the terms "bibliometric" and "Africa". The search revealed 15 bibliometric studies with data on Africa [16-30]. These were either studies that had a global reach but reported data on Africa [19-29] or studies that focused on one or more African countries [16-18,30]. None of these was a bibliometric study of childhood immunization research.

Rahman and Fukui studied factors related to worldwide variation in biomedical research productivity, and found that gross national product per capita and research and development expenditure were significant determinants of biomedical research productivity [37,38]. We did not find these factors to be significant predictors of immunization research productivity in Africa. The most significant predictor of immunization research productivity in Africa was found to be out-of-pocket health expenditure. The latter refers to the sum of money spent on health by private entities, such as households, commercial or mutual health insurance, non-profit institutions serving households, and resident corporations with a health services delivery or financing function. Out-of-pocket health expenditure also includes gratuities and in-kind payments to health practitioners and suppliers of goods and services whose primary intent is to contribute to the restoration or enhancement of the health status of individuals or population groups. In addition, we confirm the findings of other authors that the contribution of authors from Africa to the global biomedical research literature is minimal [18-20,22,23]. This meager biomedical research literature from the continent appears to be dominated by non-communicable disease research [25].

Locally-relevant health research is needed to ensure the effectiveness, efficiency and equity of immunization policies in Africa [14,39]. In general, health research helps to answer questions, to generate the evidence required to guide policy and to identify new tools. A descriptive analysis of study types, quality and outcomes was beyond the scope of our bibliometric analysis. However, in a related study, Machingaidze and colleagues conducted a detailed descriptive analysis of 881 childhood immunization research publications from Africa between 1970 and 2010 [40]. The studies were classified as clinical (n = 442, 50.2%) or operational (n = 439) research. Among clinical research studies, 41% were phase 1 to 4 controlled trials, 23% were burden of disease or epidemiology and 36% were other clinical studies. Among studies classified as operational research, 76% were on program management, 19% on immunization policy issues and 5% related to vaccine financing [40]. There is clearly a need for increased immunization research productivity from Africa, especially locally-relevant operational research. During the new decade of vaccines [41,42], African countries should prioritize research capacity development in vaccinology. In general, Africa requires strong leadership and political commitment, going forward, in the development of research capacity on the continent.

PubMed has been widely used for bibliometric analyses, but it is important to note that the database is dominated by English-language journals, therefore, possibly contributing to selection bias due to language barriers. By using the author addresses listed in the by-lines of research articles, one can only identify countries and organizations where the authors were employed when the research was done or where the article was written, or both. These limitations notwithstanding, we believe that this study is a good reflection of research productivity in the field of childhood immunization in Africa.

## Conclusions

There is an enormous range of locally-relevant research that could be undertaken to support routine childhood immunization programs in Africa [12,14]. This would inform the development of strategic immunization plans whose implementation will raise immunization coverage and prevent disease, disability and death on the continent [12,14]. This bibliometric study examined almost four decades of childhood immunization research production by authors from Africa. The results of the study show that the five most productive countries, in terms of absolute number of publications indexed by PubMed from 1974 to 2010, are South Africa, Nigeria, The Gambia, Egypt and Kenya. Based on the best possible estimate, the most significant determinant of immunization research productivity in Africa is private health expenditure. The lack of association between research productivity and immunization coverage may be an indication of the lack of interactive communication between health decision-makers, programme managers and researchers, to ensure that immunization policies and plans are always informed by the best available evidence.

## Abbreviations

BIC: Bayesian Information Criterion; CIs: confidence intervals; DTP3: third dose of diphtheria: tetanus and pertussis vaccine; EPI: Expanded Program on Immunization; GDP: gross domestic product; IRR: incidence rate ratios; WHO: World Health Organization

## Competing interests

The authors declare that they have no competing interests.

## Authors' contributions

All authors participated in discussions about the data source and planning of the analyses, and critically revised successive versions of the paper. CSW conceived the study, and CSW and OAU did the analyses and prepared the first draft of the paper. All authors have seen and approved the final version of the paper.

## Pre-publication history

The pre-publication history for this paper can be accessed here:

http://www.biomedcentral.com/1741-7015/11/66/prepub

## Supplementary Material

Additional file 1 **Comparison of models for goodness-of-fit**. In this additional file we describe the tests we conducted to investigate which regression model provides the best fit for the empirical publication data on childhood immunization research volume from Africa, including probability distributions and Bayesian Information Criterion. Click here for file
