# Supplementary material for: A bibliometric analysis of childhood immunization research productivity in Africa since the onset of the Expanded Program on Immunization in 1974
Source: BMC Med. 2013 Mar 14;11:66. doi: 10.1186/1741-7015-11-66 (PMC3599719; doi:10.1186/1741-7015-11-66)
Supplement: Additional file 1 — Comparison of models for goodness-of-fit. In this additional file we describe the tests we conducted to investigate which regression model provides the best fit for the empirical publication data on childhood immunization research volume from Africa, including probability distributions and Bayesian Information Criterion. [file 1741-7015-11-66-S1.DOC]

We fitted Poisson, negative binomial, zero-truncated Poisson, and zero-truncated negative binomial regression models for count outcomes to the number of immunisation articles indexed in PubMed so as to examine and compare which model provides the best fit for the empirical data. Analyses were run using Stata version 12 for Windows. Firstly, we examined the probability distributions which underpin the models to see how best they fit the observed data. In order to do this, the models were run without any covariates i.e. from models with intercept only. Secondly, regression models were then fitted with covariates. To obtain a graphical illustration of fits (intercept only and adjusted models), the observed minus predicted probabilities across models were plotted (Additional Figure 1). Bayesian Information Criterion (BIC) was then used to formally compare the regression models, in terms of the goodness of their fit to the empirical publication count (Additional tables 1 and 2).

***Graphical illustrations***

Additional Figure 1A shows how the probability distributions, which underpin the regression models, fit the observed number of articles indexed in PubMed. Points above zero on the y-axis indicate under-prediction, more observed counts than predicted. Whereas those below zero indicated over-prediction, more predicted counts than observed. The Poisson and zero-truncated Poisson models were poor fits, whereas negative binomial and zero-truncated negative binomial models were very close to the observed. Additional Figure 1B shows the observed minus predicted probabilities at each publication count for the covariates adjusted model. It is clear then that the zero-truncated Poisson does not predict the data well. The zero-truncated negative binomial model is a substantial improvement over the zero-truncated Poisson.

**Additional Figure 1: Observed minus predicted probabilities for the four models considered**

**
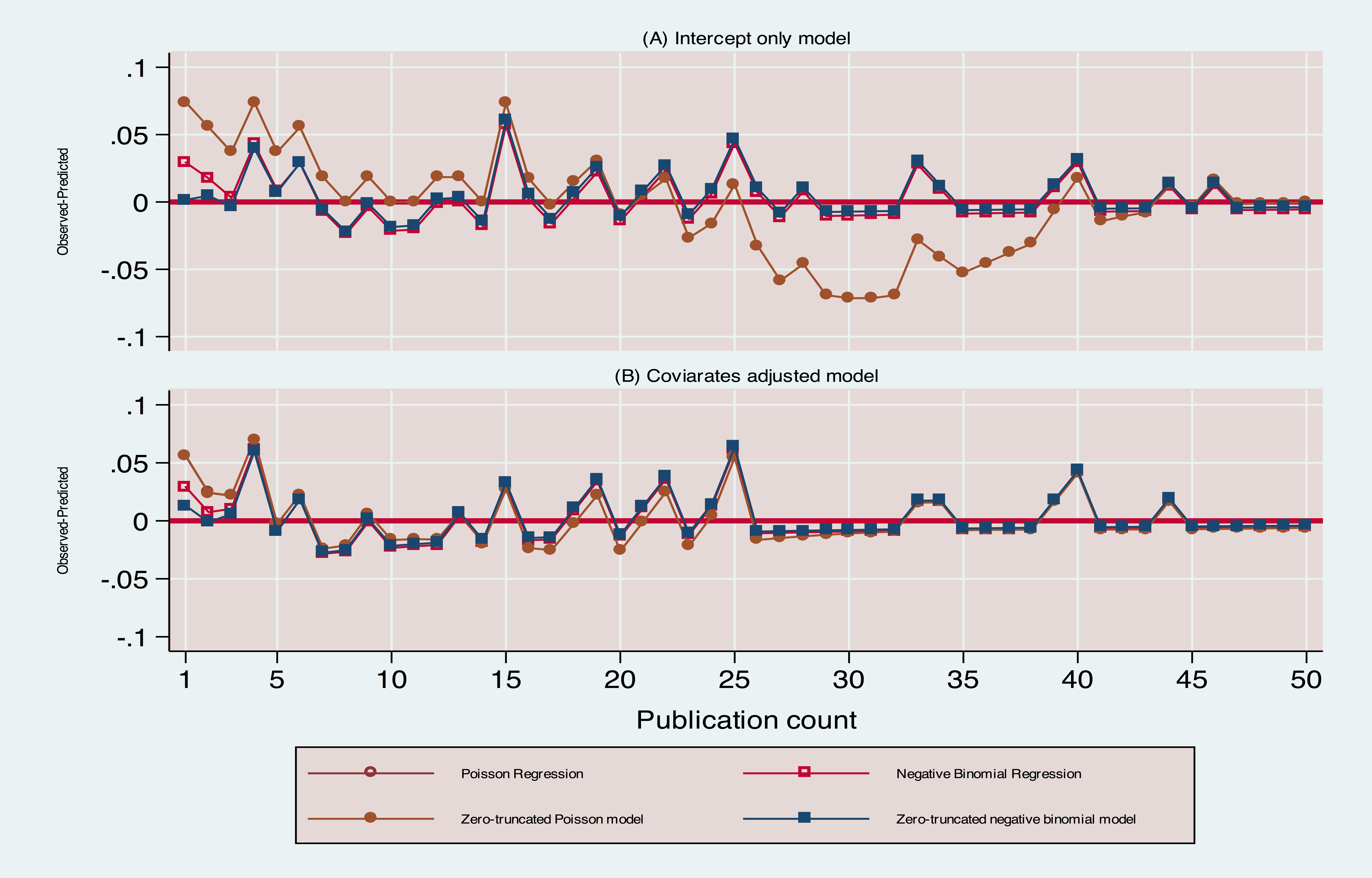
**

**Model fit and comparison test**

In order to help assess how well the Poisson model fits the data, we conducted Pearson’s and Deviation goodness-of-fit tests (Additional table 1).

Additional Table 1: Results of Poisson’s goodness-of-fit tests

| Deviance goodness-of-fit = 924.3724  Prob > chi2(35) = 0.0000  Pearson goodness-of-fit = 2127.541  Prob > chi2(35) = 0.0000 |
| --- |

Based on the results above, we concluded that the Poisson model did not fit the model well, because the goodness-of-fit chi-squared tests were statistically significant. As Additioal Table 2 shows, zero-truncated negative binomial regression provided the best fit to the data because the BIC was lowest.

**Additional Table 2: Bayesian Information Criterion for the models considered**

| **Model** | **Number of observations (N)** | **Number of parameters (K)** | **BIC** |
| --- | --- | --- | --- |
| Poisson | 41 | 6 | 1133.21 |
| ZTP | 41 | 6 | 1132.64 |
| Negative binomial | 41 | 7 | 372.21 |
| ZTNB | 41 | 7 | 367.21 |

BIC, Bayesian Information Criterion; ZTP, Zero-truncated Poisson; ZTNB, Zero-truncated negative binomial.

**Best-fit regression model**

Based on graphical methods (Additional Figure 1) and formal tests (Additional tables 1 and 2), the zero-truncated negative binomial regression model is the most parsimonious model which provides the best fit to the observed data.
